# Ambivalence, universality, and collectives—Three questions for recognition theory

**DOI:** 10.3389/fsoc.2026.1686451

**Published:** 2026-02-12

**Authors:** Onni Hirvonen

**Affiliations:** Department of Social Sciences and Philosophy, University of Jyväskylä, Jyväskylä, Finland

**Keywords:** ambivalence, critical theory, historicity, institutions, normative grounding, recognition, universality

## Abstract

This text outlines three interlinked questions for contemporary recognition theory: (a) the question of ambivalence, (b) the question of universality/historicity, and (c) the question of collective recognition. It is argued that all the questions present “forks” in the theoretical road, providing two or more distinct pathways forward. Different responses to these questions lead to three distinct approaches to recognition theory and, consequently, three different interpretations of recognition theory as a critical theory: (1) an anthropologically grounded universal theory of recognition; (2) an institutionally mediated universal theory of recognition; or (3) a localized and historicized theory of recognition. The article concludes that a deeper analysis of the institutional mediation of recognition and recognition institutions is required to clarify the differences between the available positions and to enable informed choices to be made between them.

## 1 Introduction

Recognition is one of the key concepts in critical social research over the past 30 years. Since the early 1990s, following largely the seminal work done by (Honneth 1995) and (Taylor 1994), contemporary recognition theory has established itself as an expansive research program in social and political philosophy. In that time, we have come far from the early Fichtean-Hegelian roots of the concept, and the contemporary recognition theories have done great work to iron out the many theoretical wrinkles and fill the lacunae left by the earlier formulations. As it currently stands, the concept of “recognition” is central for explaining agents' motivations and seeing what drives social action, thereby showing why interpersonal relations matter so much. Recognition is commonly understood as a personal and political concept, which draws from deep psychological needs and human interaction, and contextualizes these in social and political life.

The theoretical framework of recognition includes, however, a number of contested themes. In this text I will explore three central and interlinked questions: (a) Whether recognition is “positive” or “ambivalent”? (b) Whether recognition is universal or localized and historical? And (c) how to conceptualize collective recognition? All the questions present “forks” in the theoretical road, providing two or more distinct pathways forward.

In this paper, I claim, first, that the answers that we give to the above three theoretical questions inform largely what sort of critical theory recognition theory is. The question of ambivalence is directly related to how binding normative direction the concept of recognition is able to provide, and whether theories of recognition need to take care to spell out the potentially hidden power relations and ideological assumptions behind recognition relationships. The question of universality/historicity is connected to the scope of recognition theory (as a critical theory): whether it functions as a universal normative theory or provides at best only a localized platform for criticism. Thirdly, the question of collective recognition focuses on the role of collectives and institutions in recognition: depending on how the concepts of collective recognition are formulated, we can get very different understandings on such issues as inter-state recognition, cultural recognition, and institutional recognition. It is argued (in Section 4) that different answers to questions (a) and (b) give rise to three alternative strands of recognition theory: (1) anthropologically grounded universal theory of recognition, (2) institutionally mediated universal theory of recognition, and (3) institutionally mediated localized theory of recognition.

My second main claim is that there is an underdeveloped area in recognition theory, which would help to provide answers to the questions (a) and (b), and enable informed choices between different strands of recognition theory. Namely, focusing more closely on the role of collective and institutional recognition (question c). Many of the answers to the central questions of recognition theory hinge on the so-called institutional mediation of recognition. Depending on, for example, how we spell out the connections between “institutional” and “anthropological” sides of recognition and whether we see recognition as necessarily institutionally mediated, the possible space of answers to the questions of ambivalence and universality/historicity is restricted. To answer the questions of institutional mediation, we need to focus on the understanding of institutional realm within recognition theory and evaluate whether the views of institutions themselves are adequately conceptualized. In short, critical theory of recognition would benefit from a deeper understanding of the institutions of recognition, and an analysis of institutions of recognition would also help to answer other central theoretical questions.

I will begin by outlining the key features of contemporary recognition theory (Section 2). I will then move onto detailing the three questions (Section 3). After that, I will discuss three distinct paths that recognition theory has taken in the past decades—partly as a response to the outlined theoretical questions—and delve into some of the challenges that each of these strands of recognition face (Section 4). The article finishes with a short summary and a discussion on the role of institutions in recognition (Section 5).

## 2 The relevance of recognition

In this section, I will provide a brief overview of the central features of contemporary recognition theory. Or, to be more exact, contemporary recognition theories, as there is no single broadly accepted recognition theory.

Recognition theories commonly place their roots in the works of Fichte and Hegel.^1^ Both authors provided early formulations on the importance of relations with others for self-consciousness. These ideas were most famously revived in the works of Taylor and Honneth in the early 1990s.^2^ Since then the Hegelian recognition has developed into a wide-branching family of philosophical and social theorizing, with dedicated focus on interpersonal attitudes and ways of relating to others.^3^ These theories hold that recognition is—to follow Taylor's famous formulation—“a vital human need” (Taylor, 1994, p. 26). The desire and need for recognition characterize the human life-form (Ikäheimo, 2022).

What makes social recognition so important? Firstly, a starting point for recognition theories is that recognition is *constitutive* of personhood and persons' self-relations. We, as social beings, need recognition to be what we are. In Hegel's (1999) formulations, self-consciousnesses need affirmation from other self-consciousnesses, whereas more contemporary theories usually frame these in terms of self-relations or identity (Honneth, 1995; Fraser and Honneth, 2003). Secondly, recognition is also *responsive* to something in the other—be it features, traits, acts, personhood, and/or self-consciousness. In other words, there are conditions of recognizability, which may of course vary. Thirdly, recognition should be *reciprocal* or *mutual*. Already in the dialectic of lord and bondsman, Hegel highlights the mutuality of the pure concept of recognition in which the different parties “*recognize* themselves as *mutually recognizing one another*” (Hegel, 1999, §19). To be successful, recognition needs to be noticed and accepted, and also reciprocated in the sense that the other is taken as a valid recognizer. Hegel's insight—that is also retained in the contemporary theories—is that recognition needs to be freely given to truly count as recognition, and that the participants need to give each other a relevant standing as a recognizer.

Two further remarks apply to most contemporary recognition theories. First, recognition is commonly taken to come in different forms or attitude complexes that target different aspects of personhood. The tripartite distinction between love, respect, and esteem, as introduced by (Honneth 1995, ch. 5), is perhaps the most well-known categorization of forms of recognition. In more abstract sense, these denote attitudes of care, equality of status and standing in the making of our normative realm, and, lastly, differentiating appraisal of merits and features (Ikäheimo, 2022, p. 14). Second, recognition theories are not merely interested in the philosophical-anthropological claims about the constitution of personhood, but they are interested in the social-political realm of struggles for recognition. In short, what recognition theories aim to provide is a normative yardstick for social criticisms, which is grounded in the human need for recognition. The *psychological* side of recognition is tied as a motivating aspect to the *political* side of recognition and social struggles for recognition. The need for social recognition can be harnessed as a political force, or, alternatively, recognition can be seen as a resource that ought to be justly distributed in a society (McBride, 2013, pp. 112–115).

To sum up, recognition is constitutive of personhood (or identity), it is responsive to some standards or potentials, it has inbuilt norms of mutuality, it comes in many forms, and ultimately, recognition is a concept that can be used for critical social analysis. However, these formulations leave plenty of room for debate and, in the next section, I expound three central places of theoretical contention.

## 3 Three questions

In this section, I outline three theoretical questions that have been raised by critics and recognition theorists alike—questions that make the recognition-based foundation for critical social theory perhaps more problematic, or at least more qualified. These are the question of ambivalence of recognition, historicity-universality question, and the question of collective recognition. All of these present “forks in the road”^4^, and choosing answers to these dilemma-like questions will shape the form that recognition theory, as a critical social theory, can take.

### 3.1 Ambivalence

The question of ambivalence concerns the worry that the early 1990s descriptions of recognition are overly optimistic and constructive. These descriptions neglect the “dark side” and the negative effects of recognition. The main question is whether there are pure (and positive) forms of recognition, which could provide a normative basis for critical theory of recognition, or whether recognition will necessarily be ideologically infused and limiting.

Drawing from a recent contribution by (Ikäheimo et al. 2021), we can outline four different senses in which ambivalence of recognition can be understood. *First*, even if recognition is understood in its core to be an affirmative constructive relation, the Hegelian notion is ambivalent in the sense that dynamics of recognition can lead to unsatisfactory, freedom-undermining, and dominating relationships and dependencies (Ikäheimo et al., 2021, p. 3). In other words, recognition-relationships are prone to fail insofar as we compare their success to the ideal state (or the pure concept) of mutual recognition. The possibility of failure is well-acknowledged in the literature. Even Hegel's early example of lord-bondsman dialectics emphasizes the possibility of failure of recognition. This also highlights the connection between recognition and power (see, e.g., van den Brink and Owen, 2003). Social relations of recognition often involve disparities of power, which in turn might skew the possibilities for ideal recognition. On the other hand, failures of recognition can result from the ambivalent nature of social struggles themselves. As (Honneth 2007, p. 77) argues, “[t]he sense of no longer being included within the network of social recognition […] lacks any normative indication or direction that would stipulate in what ways one should struggle against the experience of disrespect and humiliation.” In other words, lack of recognition can motivate struggles that—even if successful—might not resolve the underlying disrespect.

*Second*, ambivalence can point to the usage of language of recognition: if social struggles are conducted under the banner of recognition, then agents might misunderstand their real—for example, redistributive—interests that would be better furthered under some other banner (Ikäheimo et al., 2021, pp. 4–5). This was the sense of “ambivalence” behind Fraser's (2000; Fraser and Honneth, 2003) criticism of Honneth's recognition theory, stating that recognition as a theoretical tool cannot grasp certain relevant aspects of social injustices. Although the debate did include some dispute over the meaning of recognition, the central disagreement concerns the scope of recognition theory and the use of recognition as an explanatory term instead of the normative ambivalence of recognition. I will set aside the first two senses of ambivalence aside here, as they are either already incorporated into recognition theory or do not exactly concern the normative ambivalence of recognition. However, there are two further senses of ambivalence that posit a deeper challenge for recognition theories.

*The third* sense of ambivalence is the claim that recognition arbitrarily fixes identities from the outside, and therefore it is, from the get-go, negative recognition, misrecognition, or ambivalent recognition (Ikäheimo et al., 2021, p. 3). This sort of negative recognition can be described from the perspective of the agent as Rousseauian inflamed desire for acceptance of one's ego—a desire for recognition that is harmful for the persons themselves and distorts social relations (Rousseau, 2012; see also Hirvonen, 2023). This is an overt desire for recognition, which, instead of supporting an independent authentic self, makes one reliant on others and their opinions. The negative of recognition could be viewed as a desire for recognition that is harmful as it locks one into a role that is externally defined and limiting.

*Fourth*, recognition can be understood as a mechanism that merely reproduces the prevailing social order and by making people to adopt specific social roles and social identities (Ikäheimo et al., p. 5). In short, the mere desire for recognition does not necessarily tell us anything about whether the social order within which it is sought is justifiable. The fact that we need it does not make all forms of recognition automatically good, as recognition orders can be twisted or ideological. As (Butler 2004, p. 3) notes, it is sometimes even better to not be recognized at all than to be recognized within a pathological system of recognition.

These last two senses of ambivalence present the biggest challenges to recognition theories: How can a critical theory that is based on recognition be successful if either the desire for recognition is already negative or the institutions of recognition are distorted? These worries concern two sides of recognition: interpersonal and institutional.

The hard Rousseauian negative claim about interpersonal recognition being negative directly contradicts the Hegelian recognition theory, which would not agree that all recognition is necessarily misrecognition (see Honneth, 2021a, pp. 141–142). Arguably, even Rousseau does not commit to the strong formulation as he also acknowledges the positive role of certain kinds of social relations (Neuhouser, 2008, p. 35). If we make the claim weaker, so that not all recognition is necessarily negative, but that recognition *can* have negative effects, we are back with the first kind of ambivalence of recognition. Here, I think that Butler manages to capture the issue well. According to her, full recognition is impossible and recognition cannot capture the full personhood of the other at once. At the same time, Butler takes seriously that the identities that are at stake with social recognition are not just clear identifications with external others or clear support for a discernible self. Rather, what happens is a “primary dislocation” (Butler, 2021, p. 35) of oneself in an ambivalent and emergent social relation. Honneth's view is set as a contrast to this. While not denying the potential external negative subjectificating effects of social relations, Honneth takes the core of Hegelian sense of recognition to be a sense of mutual normative-status-granting or authority-giving, which transforms the participants in the relation (Honneth, 2021b, pp. 24–25). This is, for Honneth, different from mere ascription of features or social statuses—it has a self-limiting *normative* function besides the mere *cognitive* ascriptive side. In short, Honnethian recognition is not about mere ascriptions of properties; ascriptions of normative statuses, which demand normative self-constraint on the part of the recognizer, are central to it (Honneth, 2021b, p. 27).

These debates are connected to the institutional side of recognition—and thus to the fourth kind of ambivalence. I will return to the role of institutional recognition later (in Section 4), but at this point, we can already anticipate that the solutions to the question of ambivalence depend largely on how we understand institutions of recognition and to what extent we see them as restricting and/or enabling recognition.

Summing up, the question of ambivalence concerns whether there are pure (and positive) forms of recognition, which could provide a normative basis for critical theory of recognition, or whether recognition will necessarily be either ideologically infused or otherwise harmful for the participants in recognition relations. For any recognition theorist worth their salt, admitting some forms of ambivalence is perhaps necessary. However, the devil lies in the details: Insofar as we accept ambivalence, what is left of the positive normative function of recognition for critical theory? What is left of the normative leverage that recognition is supposed to bring?

### 3.2 Universality/historicity

Another central debate surrounds the question whether recognition is an anthropological constant, tied to human nature, or is it something that shifts and changes between cultures and times? On the one hand, recognition is commonly described as an institutionally mediated normative response to others. It has historically developed forms that are tied to the institutions of a particular time (e.g., family, markets, civil society). On the other hand, recognition is seen as a universal human need, a feature of human life-form. It is something that is an essential part of our lives as humans and extends beyond its historical and localized realizations. The ongoing debate concerns how (and if) the anthropologically grounded forms of recognition inform the more localized cultural or identity-political struggles for recognition, and how they fit together with the fact of plurality and differing views of the good life. Furthermore, this question concerns the universalizability of recognition theory. A shared philosophical-anthropological basis of recognition could perhaps provide a recognition theory that is relevant beyond particularized social contexts, whereas a strongly historicized recognition theory might not be able extend its normative insights beyond the immediate context.

As an example, Honneth is on the fence in this matter. Giving support to universalized reading, he states that the need for social recognition is “quasi-transcendental” (Honneth, 2003, p. 174). However, he does also rely on the idea that recognition is realized, represented, and manifested in social or institutional spheres. As (Anderson 2013, p. 18) summarizes, in Honneth's model:

“(1) Humans have an historically conditioned but anthropologically grounded need for relations of mutual recognition and the associated forms of social freedom. (2) These recognition relations are in turn dependent on something like a socio-cultural ecosystem…”

In the Honnethian oeuvre (parallel to Taylor's view), the different forms of recognition become distinguished from each other through the historical development of modern society and the separation of its institutional spheres (i.e., family, markets, and civil society). These in turn reflect, realize, and express different forms of recognition. (Deranty 2022) specifically emphasizes the historical side of Honneth's critical project, claiming that immanent critique based on recognition is and should be limited to its context.

However, it is contested how much role we should give to the socio-cultural ecosystem or the institutional realizations of recognition relationships. In a contrast to the Honnethian view, Ikäheimo defends a universalist model of recognition in order to save the relevance of recognition for the whole human life-form. His worry is that if we tie recognition too tightly together with a specific institutional setting, it will appear relevant only for that particular social setting. “[I]f the recognition principles really are specific to European modernity or the capitalist-bourgeois social order, and if social critique has to appeal to these and no other principles in order to be immanent,” then recognition theory is “irrelevant for the largest part of humanity” (Ikäheimo, 2022, p. 168).

Ikäheimo sees the institutionally mediated forms of recognition as secondary to what he refers to as purely intersubjective modes of recognition related to human species-being. What is at stake here is, first, the normative grounding of recognition theory: is it based on universally shared needs of recognition or are the normative grounds immanent to social institutions? Secondly, as mentioned above, the question concerns the universalizability of recognition claims or the scope of the critical potential of recognition theory. Ikäheimo's universalism and Honneth's account of historically developed institutionally mediated recognition are, of course, not the only available options. Diverging partly from these options is, for example, McBride's (2013) suggestion, that we need a complementary—potentially more universalistic—theory of justice, as recognition theory cannot provide such grounds by itself. In a sense, recognition can be seen as a resource, but the criteria for its sharing should and could draw from a broader normative framework of justice.^5^

Again, we are presented with a theoretical choice that offers very different outlooks for a critical theory of recognition. As with ambivalence, I suggest that we cannot sufficiently answer the question of the universality of recognition without first clarifying what institutional recognition or institutional mediation of recognition entails. To accept, deny, or diminish the role of institutional recognition, we must have a clear understanding of what is at stake when we talk about, for example, recognition by institutions, institutional role-expectations, or recognition of institutions, and so forth. This, in turn, is related to the role of collective recognition in general.

### 3.3 Collective recognition

Although the key definitions of recognition theory focus on the dynamics of interpersonal recognition between individual persons, recognition theory as a social and political theory has also found its use in collective contexts. The paradigmatic cases being the different social movements, as well as international politics, construed as a struggle for recognition between states. The contemporary political theories of recognition make the shift from individual dynamics of recognition into the collective contexts of recognition usually without much theoretical deliberation. However, it is questionable whether the concept of recognition allows shifting from individuals' interactions to collective action without complications.

Against these doubts, recognition has been seen as a helpful concept for analyzing the dynamics of collective action and group agency as well as our relations to groups (see e.g., Lindemann and Ringmar, 2012; Hirvonen, 2017). It forces us to focus on the forms of the relations, the relevant agent-positions in these relations (individual–individual, group–group, individual–group, membership), and the enabling conditions of those relations. Furthermore, the picture gets more complicated if recognitive attitudes are understood as dependent on the institutional setting—mediated by social structures and norms. Consequently, there is a pressing need to spell out the details of collective and institutional recognition. Unlike the previous questions, which are central parts of major debates in the field, the question of collective recognition is less acknowledged in the current literature. Here, my aim is to spell out the central themes and reinforce the view that proper analysis of institutional recognition in fact holds the key for answering the central theoretical questions of recognition theory.

Drawing from Hegel, we can already see a tension in the usage of the term recognition in collective contexts. In *Phenomenology of Spirit*, (Hegel 1999) had described a struggle between two self-consciousnesses who must realize that, instead of turning into a dominating relationship of lord and bondsman, recognition must be freely given and mutual. As also argued in contemporary theories, this kind of social affirmation is what makes us individuals or persons. In *Philosophy of Right*, Hegel extends this thought of necessity of recognition to states as individual agents:

“Just as the individual person is not real unless related to others [...] so the state is not really individual unless related to other states.” (Hegel, 2000, §331)

However, Hegel already understands that the comparison is problematic. First, states are not similarly dependent on others as individuals are:

“individuals are dependent upon one another in a great variety of ways, while independent states are wholes, which find satisfaction in the main within themselves.” (Hegel, 2000, §332)

A state is a self-sufficient “completely independent totality” (Hegel, 2000, §330) and thus, the dynamics of recognition work differently than in the case of dependent individuals, who have to come to the realization that they need others. More generally, and not just in terms of states, collective agents can differ significantly from individual agents, and recognition theories need to be perceptive of these differences.

Although Hegel himself appreciates the complications of collective recognition, he does not discuss the collectivization of recognition in detail. From a contemporary perspective, recognition theory should be sharpened by careful analysis and distinction of the conceptual elements related to collective recognition. First, we should distinguish collective recognition from institutional mediation of recognition, or recognition institutions. In collective recognition, collective entities function as agents of recognition, whereas institutional mediation of recognition refers to a normative framework (i.e., the institution). Indeed, institutional recognition seems to have two senses: sometimes institutions are taken as entities who are recognizing and are recognized; sometimes institutions refer to normative frameworks that enable and guide recognition. In short, institutions might take agential roles in recognition, or they might provide a background for recognition—and these two senses present very different but not exclusive senses of institution.

In what follows, I will go through three distinct approaches to recognition theory that solve the questions of ambivalence and universalism differently. I also propose that solving the question of collective and institutional recognition will help solve the questions of ambivalence and historicity. However, the question of collective recognition presents its own fork in the theoretical road. On the one hand, there is the question of group agency in recognition: Should recognition be understood as something that fundamentally happens at an individual level (a singularist position), or should we include group agents and terms such as vertical recognition toward groups and institutions in our theorization (a non-singularist position)? These are questions that need social-ontological analysis. On the other hand, the question of institutional mediation and its relation to potential purely intersubjective forms of recognition remains unsolved. The suggestion here is that having an informed view of institutional recognition will make actual disagreements in other areas more visible, while ensuring conceptual consistency.

## 4 Three strands of recognition theory

The questions of ambivalence and universality of recognition are closely intertwined. They both concern whether recognition can be used as a yardstick for justice, and if so, in what contexts. Answers to this rest partly on the answer to the question of how we understand the role of institutional mediation of recognition. We should address the theoretical question of whether recognition is necessarily institutionally mediated, or if there are purely intersubjective forms of recognition that could offer a way out of institutionally biased or ideological recognition. If we accept that recognition is necessarily institutionally mediated, then the question of the critical potential of recognition theory becomes more acute. In short, if the concept of recognition is to have any emancipatory potential, then there needs to be some way of preserving the anti-ideological and non-determined moments of recognition. These options present three different paths for a theory of recognition, three forks in the road: either (1) an anthropologically grounded or (2) an institutionally mediated universal theory of recognition or (3) a localized and historicized theory of recognition. Next, I briefly discuss all three options, outlining the central features of each of these paths.

### 4.1 Anthropologically grounded universal recognition

In this view, recognition gets its normative grounding from the shared human nature. This solution aligns with universalist claims about recognition: Since recognition is a central part of the human life-form, there is a shared natural standard for recognition. This can be used as grounds to separate ambivalent forms of recognition from the purely intersubjective (see Ikäheimo, 2022, p. 20) and positive forms of recognition. This is a form of recognition theory that is happy to bite the bullet with regard to essentialism. Indeed, the newly proposed critical naturalism (e.g., Gregoratto et al., 2022) is parallel to this project of recognition theory, which seeks to extract normativity from shared human nature. For example, Ikäheimo's (2022, p. 195) recognition theory sees the question of natural normativity as the most central question, and he seeks to draw norms of recognition from philosophical anthropology in order to build a universal normative framework that is relevant for all human persons and all cultures.

There are multiple difficult challenges for this strand of recognition theory. The first is obvious: It needs to sidestep the naturalist fallacy and explain how natural normativity arises. How do strong normative forms of recognition arise from nature, especially if we abstract from cultural and institutional aspects of recognition? Anthropologically grounded theory seems to need an accompanying “natural rights” theory of recognition. The second challenge is connected to holism: It can be doubted whether purely intersubjective recognition can be meaningful without any broader shared horizon of meanings. Norm-guided social practices might be necessary for making sense of any interpersonal recognition.

Assuming these problems can be solved, there is also a challenge from the institutional side of recognition. First, the connections between purely intersubjective and institutional recognition need to be explained. For example, if institutions reflect interpersonal recognitive attitudes, how can there be a disconnect between purely intersubjective and institutional recognition? Second, institutions and institutional recognition are also important from the social-theoretical side, as they are the focus of critical social theory, which looks to diagnose social pathologies and foster institutional change. In short, institutional recognition is a central part of any theory of recognition—even if its normative core would be derived from universal anthropological grounds.

### 4.2 Institutionally mediated universal recognition

The following two options (Sections 4.2 and 4.3) embrace the idea that recognition is necessarily institutionally mediated.^6^ However, they differ in their view of how universalizable the normative core of recognition is.

With these views, even the most basic recognition needs can be interpreted as dependent on institutions. This, in turn, leaves the door open for ambivalence of recognition: not all historically formed ways of realizing recognition are affirmative or positive. In the pessimistic Althusserian interpretation, any institution is ideological and geared toward upholding the status quo (Honneth, 2021a, pp. 49–50). However, critical theorists commonly want to leave theoretical room for social transformation—partly because, without it, the whole critical project would be futile, and partly because social change does actually occur. For example, (Butler 2021, p. 39) wants to warn against the rigid and constraining recognition categories, but she does not deem ideological recognition orders to be as deterministic that they would snuff out all possibilities of agency.

“We do not make up those terms through which we are recognized since we are born into a language that precedes us and acts on us. But neither are we fully determined by such discourses; they can be resisted, resignified, dissolved, and new language can be wrought from and against the old.” (Butler, 2021, p. 44)

In short, institution-based recognition theories require a social ontology of institutions that allows for change. This would, ideally, include elements from social sciences, but also from social ontology of institutions and normative orders.^7^ This is not to claim that social ontologists understand things correctly right now, but rather that critical theories of recognition that are based on institutionally mediated recognition need an accurate account of institutions and institutional change. This should not be a problem, since any true account of institutions will account for institutional change. Here social ontology can function as an extra tool in the already multidisciplinary toolbox of critical theorists.

These considerations apply to any institutionally mediated recognition. However, there are two separate strands that institution-based recognition theory can take, and they differ in their answers to the universality/historicity question. The first option looks to globalize and universalize recognition, despite its necessary connections to institutional reality. The second option abstains from universalization altogether (see Section 4.3). There are at least two strategies for achieving universalization. First, universalization can be seen as a positive historical trajectory of recognition institutions. For example, in Honneth's account, moral progress is tied to expanding the spheres of recognition (Honneth, 1995; see also Honneth, 2002). Honneth's theory gives a backwards-looking, historically developing standard for universalizing the normative core of recognition theory. Indeed, he aims to give a universal normative grammar of social conflicts through his recognition theory. However, this kind of theory does still struggle with the question of ambivalence, especially in cases of reconciling contradictory spheres of recognition.^8^

The second strategy of universalization is the already mentioned view that sees recognition more as a resource and complements recognition theories with other universal theories of justice (e.g., McBride, 2013), which handle the normative grounds of distributing recognition. Although this view sees recognition as an important element of human life, it does not base its critical normative claims on recognition alone, and as such it relegates recognition to a secondary role in critical social theory.

### 4.3 Institutionally mediated localized recognition

The third road that recognition theory can take is perhaps the most faithful to the project of immanent critique. According to it, critical theory of recognition is organically related to its social and political context, and internal to it (Deranty, 2022, p. 178). The universal extension of norms of recognition is unnecessary and futile, as they are necessarily tied to their local institutional context. This does not mean that the normative insights of recognition theory would not be translatable from one context to another; rather, the theory's aim is not to provide a universal moral framework, but to focus on more localized and historical recognition practices. As (Bernstein 2022) notes in his critique of anthropologically grounded universal recognition, the whole idea of unconditional universal recognition is a modern invention.

This kind of approach is open to accusations of relativism and ambivalence. Thus, perhaps the greatest challenge for the localized recognition theory is to provide convincing normative grounds for criticism—even in a particularized context. It cannot rely on universal moral theory or shared anthropology. There are, of course, different strategies for achieving this. Two of them can be gleaned from Honneth's work: normative reconstruction and a focus on social suffering. The former aims to provide an empirically grounded historical interpretation of particular institutions and their normative commitments that can be used as grounds for criticising for those institutions (Honneth, 2014). This approach also requires a particular social ontology that views institutions as entities based on normative promises and commitments. The latter approach takes a page from anthropological (or psychological) book and aims to ground normative demands in experienced, socially caused suffering. From this perspective recognition theory is a negative theory that focuses on social pathologies and does not provide a perfectionist view of a good society (Renault, 2002; Zurn, 2000, p. 118). Here, of course, theoretical questions arise as well because the inchoate feelings of lack of recognition need to be connected to the institutional realm and social causes. The question of ideological recognition (ambivalence) is especially relevant here, as it can be argued that not all feelings of a lack of recognition are normatively equal nor do they demand actions from a perspective of justice. Consider, for example, the prevalent middle-class fear of losing their privileged social position. While this is a genuine concern, but it would be a stretch from the perspective of justice to argue that privileged positions should be upheld merely on the basis of the feelings of privileged groups. The third option is to view relativism as a theoretical strength rather than a weakness: Universalizing pretensions should be in fact dismantled, and the theory should embrace the idea that there are different localized ways of being human and being recognized.

## 5 In conclusion: on institutions of recognition

As is typical with critical social theory, there are different theoretical strands within recognition theory, pulling in different directions. In a sense, recognition theory is and has always been at a crossroads: How positive or negative understanding of recognition do we have? How anthropological or institutional recognition is? The answers to these questions have a massive impact on the shape that a critical theory of recognition can take. Recognition could be based on anthropological constants that are universal to humans (Ikäheimo); it could be institutionally mediated and on a historical trajectory toward universalization (Honneth); it could be universalized with a complementary theory of justice (McBride); or one could accept the limited immanent scope of recognition-based critical theory (Deranty).

The above discussion also suggests a method for clarifying the differences between these positions and choosing among them: namely, an analysis of recognition institutions. This task involves answering two separate big questions. First, there is the general question of what institutions are, and what sort of institutions are at stake with recognition. Second, there is the question of the institutional mediation of recognition and its relation to potential non-institutional “purely intersubjective” forms of recognition.

When institutions figure in recognition theories, what is it that we are describing? Here recognition theorists can draw from contemporary social ontology, which includes many detailed accounts of the institutional realm. Raimo Tuomela's general taxonomy is a helpful starting point. For him, institutions are social-normative systems or, in his own terms, “public norm-practice systems” (Tuomela, 2013, p. 218). These normative systems come in four forms. (A) In the broadest sense, institution means a norm-governed social practice. (B) Institutions can be understood as social systems that confer new conceptual and social statuses to some entities. (C) Institutions can be understood as conferring new deontic statuses and status functions in a collective. (D) An institution can be an organization with specific institutional roles, social positions, and, in Tuomela's parlance, a task-right system (Tuomela, 2007, pp. 196–197). The forms of institutions in the taxonomy are not exclusive, and the more structured forms of institutions build on the earlier ones and may presume some broader institutions within which they are nested. A general norm-governed phenomenon such as language or money can be an institution, as can social organizations or corporations. All are normative social orders with their own structures and rules. Some are more fluid and informal, whereas entities like corporations can be more strictly defined with regard to their purpose as well as their institutional roles and tasks.

What does this taxonomy say about recognition institutions? One implication is that there is no single sense of an institution. Recognition theorists must make it clear what type of institution they are referring to when using the term. Additionally, there is no accepted singular view of what recognition institutions are or what (institutional) mediation means. To start with the latter, mediation can mean at least two things. First, it can refer to a situation in which a distinct third party functions as a mediator between a recognizer and a recognizee, “acting as an intermediary element or agent in bringing about recognition between other two distinct parties” (Koskinen, 2019, p. 38). The second, and perhaps more common, sense of mediation is the institutional mediation of recognition where institutions instruct or even determine what counts as recognition and what counts as recognition-worthy.^9^ For example, in Honneth's view, there are “recognition orders” from which individuals draw in the course of recognition, “because it is only in light of these institutions that they grant each other a normative status” (Honneth, 2011, p. 403). However, institutions do not strictly determine or constitute recognition as they are also expressions of interpersonal relations (Deranty, 2009, p. 232). In this sense, institutions are partly constitutive of recognition (by directing it and giving it cultural forms) and partly constituted by it (through a dependence on individuals' attitudes).

These kinds of recognition institutions fit Tuomela's categories (B) and (C). They are normative frameworks that confer social statuses, standing, and powers and responsibilities. Institutions of recognition are not usually understood to represent a structured corporate form (D), but rather, they work as a cultural ecosystem that orders social action and makes certain kinds of social actions sensible. Additionally, recognition theories can consider institutions and groups in agential roles, as agents that give and receive recognition. However, these institutions or groups are clearly very different from institutions as normative frameworks.

Here I cannot provide a theory of institutions that would neatly fit with various theories of recognition, nor can clarify how various theories of recognition discuss institutions. However, the point of mentioning Tuomela's taxonomy is to highlight that there are relevantly different senses of institution at play, and social theories ought to be sensitive to these differences when discussing the recognition of institutions or the institutional mediation of recognition. It seems to me that one of the central future projects for recognition theory is to explicate the interconnectedness of individuals' needs for recognition (the anthropological side) and the various elements of the institutional realm. As anticipated by (Renault 2010, p. 236), a social-ontologically informed critical social theory requires an argument that can draw from both, philosophical anthropological grounds (explaining why certain forms of interpersonal action and institutions are necessary for humans) and social ontological grounds (explaining how institutions function). Once both of these elements are properly explicated, making difference between positive and ambivalent forms of recognition becomes easier and the scope of recognition-based social criticism is clarified.

## Footnotes

1 For a broader and earlier historical context, see (Saarinen 2016).

2 Although Taylor and Honneth are commonly referred to as authors who kickstarted the contemporary interest in recognition, there is of course an earlier interest in recognition as well [see, e.g., (Siep 1979) who worked on recognition right before it became widely known] that runs from Fichte and Hegel to here and now.

3 In everyday language, recognition can have different meanings, such as identification (of things or persons), recognition as acknowledgment of normative frameworks, or recognition as remembering (see Ikäheimo and Laitinen, 2007; Ricoeur, 2005). These meanings are distinct from Hegelian recognition.

4 The term is taken from Jean-Philippe Deranty's excellent analysis of Honneth's earlier work (Deranty, 2009), in which Deranty sees Honneth as navigating such forks in the road regarding the tradition of critical theory.

5 See also (Piroddi and Vuori 2025) for an argument that recognition functions as capital.

6 The whole ontologizing of recognition and forgetfulness of historicity that are suggested by the option 4.1 are argued to be a problem of recognition theory (McNay, 2021, pp. 71–74).

7 This is a brand of research that has been done externally to theories of recognition. For promising leads that could be combined with recognition theory, see, for example, (Bicchieri 2017) on the transformations of normative orders.

8 This resembles the challenges of multicultural recognition and fusion of (recognition) cultures that (Taylor 1994) raised in his essay on politics of recognition. The Honnethian approach has been criticized for underplaying the contradictions of social life (McNay, 2021, p. 76).

9 Conceptual mediation could be understood as a third sense of mediation. However, here I take the conceptualizations to be related to institutions—ultimately at least in Tuomela's broadest sense (A).

## References

[B1] Anderson J. (2013). The fragile accomplishment of social freedom. Krisis: J. Contemp. Philos. 1, 18–22. Available online at: https://krisis.eu/article/view/38983 (Accessed February 3, 2026).

[B2] Bernstein J. (2022). Review: recognition and the human life-form: beyond identity and difference. Notre Dame Philos. Rev. Available online at: ndpr.nd.edu/reviews/recognition-and-the-human-life-form-beyond-identity-and-difference/ (Accessed August 18, 2025).

[B3] Bicchieri C. (2017). Norms in the Wild: How to Diagnose, Measure, and Change Social Norms. New York, NY: Oxford Academic.

[B4] Butler J. (2004). Undoing Gender. New York, NY: Routledge.

[B5] Butler J. (2021). “Recognition and the social bond,” in Recognition and Ambivalence, eds. H. Ikäheimo, K. Lepold, and T. Stahl (New York, NY: Columbia University Press), 31–53.

[B6] Deranty J.-P. (2009). Beyond Communication: A Critical Study of Axel Honneth's Social Philosophy. Boston, MA: Brill.

[B7] Deranty J.-P. (2022). Recognition in a historical key: Axel Honneth on the history of recognition and social freedom. J. Soc. Political Philos. 1, 169–185. doi: 10.3366/jspp.2022.0024

[B8] Fraser N. (2000). Rethinking recognition. New Left Rev. 3, 107–120. doi: 10.64590/pup

[B9] Fraser N. Honneth A. (2003). Redistribution or Recognition? A Political-Philosophical Exchange. eds. N. Fraser and A. Honneth. Transl. by J. Gold, J. Ingram and C. Wilke. London: Verso.

[B10] Gregoratto F. Ikäheimo H. Renault E. Särkelä A. Testa I. (2022). Critical naturalism: a manifesto. Krisis: J. Contemp. Philos. 42, 108–124. doi: 10.21827/krisis.42.1.38637

[B11] Hegel G. W. F. (1999). “Chapter IV: the truth of self-certainty,” in Hegel's Phenomenology of Self-consciousness, eds. L. Rauch and D. Sherman (Albany, NY: SUNY Press), 13–42.

[B12] Hegel G. W. F. (2000). Philosophy of Right. Transl. by S. W. Dyde. Kitchener: Batoche Books.

[B13] Hirvonen O. (2017). Groups as persons? A suggestion for a Hegelian turn. J. Soc. Ontol. 3, 143–165. doi: 10.1515/jso-2016-0019

[B14] Hirvonen O. (2023). “The ambivalence of human sociality: Rousseau and recognition,” in Rousseau Today: Political Philosophy and Public Purpose, Eds. N. Harris, D. Bosseau, P. Pintobtang, and O. Brown (Cham: Palgrave Macmillan), 107–126.

[B15] Honneth A. (1995). The Struggle for Recognition: The Moral Grammar of Social Conflicts. Transl. by J. Anderson. Cambridge, MA: The MIT Press.

[B16] Honneth A. (2002). Grounding recognition: A rejoinder to critical questions. *Inquiry: An Interdisciplinary Journal of Philosophy*. 45, 499–519. doi: 10.1080/002017402320947577

[B17] Honneth A. (2003). “Redistribution as recognition: a response to Nancy Fraser,” in Redistribution or Recognition? A Political-Philosophical Exchange, Eds. N. Fraser and A. Honneth. Transl. by J. Gold, J. Ingram and C. Wilke (London: Verso), 110–197.

[B18] Honneth A. (2007). Disrespect: The Normative Foundations of Critical Theory. Cambridge: Polity Press.

[B19] Honneth A. (2011). “Rejoinder,” in Axel Honneth: Critical Essays, With a Reply by Axel Honneth, Ed. D. Petherbridge (Leiden/Boston, MA: Brill), 391–421.

[B20] Honneth A. (2014). Freedom's Right: The Social Foundations of Democratic Life. Cambridge: Polity Press.

[B21] Honneth A. (2021a). Recognition: A Chapter in the History of European Ideas. Cambridge: Cambridge University Press.

[B22] Honneth A. (2021b). “Recognition between power and normativity: a Hegelian critique of Judith Butler,” in Recognition and Ambivalence, Eds. H. Ikäheimo, K. Lepold, and T. Stahl (New York, NY: Columbia University Press), 21–30.

[B23] Ikäheimo H. (2022). Recognition and the Human Life-Form: Beyond Identity and Difference. New York, NY: Routledge.

[B24] Ikäheimo H. Laitinen A. (2007). “Analyzing recognition: identification, acknowledgement, and recognitive attitudes towards persons,” in Recognition and Power: Axel Honneth and the Tradition of Critical Social Theory, Eds. B. Van Den Brink and D. Owen (Cambridge: Cambridge University Press), 33–56.

[B25] Ikäheimo H. Lepold K. Stahl T. (2021). “Introduction,” in Recognition and Ambivalence, Eds. H. Ikäheimo, K. Lepold, and T. Stahl (New York, NY: Columbia University Press), 1–20.

[B26] Koskinen H. (2019). “Mediated recognition: suggestions towards an articulation,” in Recognition and Religion: Contemporary and Historical Perspectives, Eds. M. Kahlos, H. J. Koskinen, and R. Palmén (London: Routledge), 34–50.

[B27] Lindemann T. Ringmar E. (Eds.) (2012). The International Politics of Recognition. Boulder, CO: Paradigm Publishers.

[B28] McBride C. (2013). Recognition. Cambridge: Polity.

[B29] McNay L. (2021). “Historicizing recognition: from ontology to teleology,” in Recognition and Ambivalence, Eds. H. Ikäheimo, K. Lepold, and T. Stahl (New York, NY: Columbia University Press), 69–97.

[B30] Neuhouser F. (2008). Rousseau's Theodicy of Self-Love: Evil, Rationality, and the Drive for Recognition. Oxford: Oxford University Press.

[B31] Piroddi C. Vuori J. (2025). Accumulating social recognition: reproduction of social inequality in late-modern societies. J. Soc. Political Philos. 4, 156–174. doi: 10.3366/jspp.2025.0113

[B32] Renault E. (2002). La philosophie critique: porte-parole de la souffrance sociale [Critical philosophy: a spokesperson for social suffering]. Mouvements 24, 21–32. French. doi: 10.3917/mouv.024.0021

[B33] Renault E. (2010). A critical theory of social suffering. *Crit. Horiz*. 11, 221–241. doi: 10.1558/crit.v11i2.221

[B34] Ricoeur P. (2005). The Course of Recognition. Cambridge, MA: Harvard University Press.

[B35] Rousseau J.-J. (2012). “Discourse on inequality,” in The Major Political Writings of Jean-Jacques Rousseau (Chicago, IL: The University of Chicago Press), 37–151.

[B36] Saarinen R. (2016). Recognition and Religion: A Historical and Systematic Study. Oxford: Oxford University Press.

[B37] Siep L. (1979). Anerkennung als Prinzip der praktischen Philosophie. Freiburg: Alber.

[B38] Taylor C. (1994). “The politics of recognition,” in Multiculturalism: Examining the Politics of Recognition, ed. A. Gutmann (Princeton, NJ: Princeton University Press), 25–74.

[B39] Tuomela R. (2007). The Philosophy of Sociality: The Shared Point of View. New York, NY: Oxford University Press.

[B40] Tuomela R. (2013). Social Ontology: Collective Intentionality and Group Agents. New York, NY: Oxford University Press.

[B41] van den Brink B. Owen D. (eds.) (2003). Recognition and Power: Axel Honneth and the Tradition of Critical Social Theory. Cambridge: Cambridge University Press.

[B42] Zurn C. (2000). Anthropology and normativity: a critique of Axel Honneth's ‘formal conception of ethical life'. Philos. Soc. Crit. 26, 115–124. doi: 10.1177/019145370002600106

